# Differential modulation of the lactisole ‘Sweet Water Taste’ by sweeteners

**DOI:** 10.1371/journal.pone.0180787

**Published:** 2017-07-10

**Authors:** Cynthia Alvarado, Danielle Nachtigal, Jay P. Slack, Barry G. Green

**Affiliations:** 1 The John B. Pierce Laboratory, New Haven, Connecticut, United States of America; 2 Givaudan Flavors Corp, Department of Science + Technology, Cincinnati, Ohio, United States of America; 3 Department of Surgery (Otolaryngology), Yale School of Medicine, New Haven, Connecticut, United States of America; Barnard College, UNITED STATES

## Abstract

Pre-exposure to taste stimuli and certain chemicals can cause water to have a taste. Here we studied further the ‘sweet water taste’ (SWT) perceived after exposure to the sweet taste inhibitor lactisole. Experiment 1 investigated an incidental observation that presenting lactisole in mixture with sucrose reduced the intensity of the SWT. The results confirmed this observation and also showed that rinsing with sucrose after lactisole could completely eliminate the SWT. The generalizability of these findings was investigated in experiment 2 by presenting 5 additional sweeteners before, during, or after exposure to lactisole. The results found with sucrose were replicated with fructose and cyclamate, but the 3 other sweeteners were less effective suppressors of the SWT, and the 2 sweeteners having the highest potency initially enhanced it. A third experiment investigated these interactions on the tongue tip and found that the lactisole SWT was perceived only when water was actively flowed across the tongue. The same experiment yielded evidence against the possibility that suppression of the SWT following exposure to sweeteners is an aftereffect of receptor activation while providing additional support for a role of sweetener potency. Collectively these results provide new evidence that complex inhibitory and excitatory interactions occur between lactisole and agonists of the sweet taste receptor TAS1R2-TAS1R3. Receptor mechanisms that may be responsible for these interactions are discussed in the context of the current model of the SWT and the possible contribution of allosteric modulation.

## Introduction

In a benchmark study published in 1964, Bartoshuk et al. [1] showed that a sour or bitter taste sometimes reported for water was associated with adaptation to sodium chloride in saliva. Other psychophysical studies later demonstrated that in humans, water can taste sweet, salty, sour, or bitter depending on the taste stimulus [2, 3], plant extract, or other chemical (e.g. [4, 5]) that precedes it. ‘Water tastes’ were therefore concluded to be aftereffects of chemical exposures that alter the excitability of gustatory receptors.

However, the receptor mechanisms responsible for these aftereffects have been slow to be identified [6]. A biochemical mechanism for the “sweet water aftertaste” of sweet taste inhibitors was not proposed until 1999 [7], and a comprehensive study of the receptor biology of the ‘sweet water taste’ (SWT) was not published until 2006. In that study, Galindo-Cuspinera et al. [8] first measured the SWT produced by the sweet taste inhibitor lactisole and high concentrations of the sweeteners saccharin and acesulfame-K, then went on to conduct *in vitro* experiments with the human G-protein-coupled (GPCR) sweet taste receptor TAS1R2-TAS1R3. The central finding of the study was that introduction of lactisole or high concentrations of saccharin and acesulfame-K cause intracellular Ca^++^ to fall below baseline levels, and that a subsequent buffer rinse reverses this effect. Consistent with an idea proposed earlier by DuBois [9], the authors concluded that lactisole and high concentrations of saccharin or acesulfame-K bind to the transmembrane domain (TMD) of TAS1R2-TAS1R3 where they function as inverse agonists [10, 11], and that sweetness is perceived as the chemicals washout and the receptor returns to its constitutively active state [12–14].

Our interest in this hypothesis grew out of a preliminary observation that mixing sucrose with lactisole tended to suppress the SWT. In the first of 3 experiments we verified this suppressive mixture effect and also found that the SWT was partially suppressed when sucrose was presented before lactisole, and was completely suppressed when sucrose was presented after lactisole. We investigated these interactions further in 2 additional experiments: Experiment 2 found that when in mixture with lactisole 5 other sweeteners also suppressed the SWT, but that the magnitude of suppression appeared to be related to sweetener potency. In fact, the 2 sweeteners with the highest potencies enhanced the SWT during the first water rinse before suppressing it over subsequent rinses. An investigation in experiment 3 of a preliminary observation that the SWT was perceived on the tongue tip when the tongue was dipped into flowing water provided evidence of the importance of water flow for the SWT as well as support for the importance of receptor potency in the suppression of SWT. The findings from all 3 experiments are discussed within the context of the current model of the SWT and the possibility that allosteric modulation, a fundamental characteristic of GCPR function [13–17], plays a significant role in the ability of sweeteners to modulate the lactisole SWT.

## Materials and methods

### Subjects

A total of 105 subjects participated in 3 experiments: 22 (14 females) in experiment 1; 20 (15 females) in experiment 2 with an additional 29 subjects (19 females) in a control condition added after completion of experiment 2; and 34 (21 females) in experiment 3. All were recruited from public postings on the Yale Medical School and Yale College campuses, were 18 to 45 years of age, gave written informed consent, and were paid to participate. The research was conducted in accordance with the principles expressed in the Declaration of Helsinki, and the research protocol was approved by the Human Investigations Committee of the Yale University IRB. All subjects were fluent English-speakers, self-reported healthy nonsmokers who had no known taste or smell disorders or deficiencies, were not pregnant, and had no lip, cheek or tongue piercings. Subjects were asked to refrain from eating or drinking foods or beverages for at least one hour prior to their scheduled session.

### Stimuli

The following chemical stimuli were tested: Experiment 1: 8 mM lactisole (Sodium 2-(4-methoxyphenoxy) propanoic acid (Chem-Impex International Inc., Wood Dale, IL, USA) and 0.56 M sucrose (Sigma-Aldrich, St Louis, MO, USA). The lactisole concentration, which is considerably higher than that used in the study by Galindo-Cuspinera et al. [8], was chosen to ensure that any differences in SWT across conditions could be reliably measured. Experiment 2: in addition to 8 mM lactisole, 5 different sweeteners were tested: 32 mM sodium cyclamate, 1.0 M fructose, 0.28 mM neohesperidin dihydrochalcone (NHDC), 1.5 mM sucralose, and 3.2 mM sodium saccharin (Sigma-Aldrich, St Louis, MO, USA). The sweetener concentrations that were selected produced approximately equal sweetness during pilot testing with laboratory personnel. Experiment 3: 20 mM lactisole was tested with 0.56 M sucrose and 0.032 mM neotame N-[N(3,3-dimethylbutyl)-L-a-aspartyl]-L-phenylalanine 1-methyl ester. The higher concentration of lactisole was selected during pilot testing to produce a SWT on the tongue tip that was similar in intensity to that produced by whole-mouth exposures in the preceding 2 experiments. In all experiments the normally acid lactisole solutions (e.g. the 8 mM solution had a pH of approximately 4.5) were buffered to pH 7.0 with NaOH, which also eliminated its weak sour/bitter taste, and all samples and rinse water (dH_2_O) were kept at 37°C.

In experiments 1 and 2, all samples were delivered in 25-ml volumes that were sipped from medicine cups. In experiment 3, all stimuli were sampled by dipping only the tongue tip into the solutions. For conditions calling for *Still* solutions, the tongue was dipped into polystyrene Fisherbrand weighing dishes (41 x 41 x 8mm Natural) containing the solutions. In conditions calling for sampling *Flowing* solutions, the tongue was dipped into the tongue bath of a Temperature-controlled Flow Gustometer (TFG) that was designed and built in the John B. Pierce Laboratory electronics and machine shop. The TFG delivers taste solutions and dH2O rinses to the tongue tip via a temperature-controlled, dual-channel system controlled by LabVIEW software. In brief, taste solutions are pumped from glass reservoirs through 2 inline Peltier heating and cooling chambers that control the temperature of solutions delivered to an interchangeable Teflon tongue bath (~ 4-ml volume). The solution flows up from the base of the bath and through a mixing screen which helps to produce a uniform (laminar) flow of taste solution over and around the submerged tongue tip. Solution depth is kept constant by allowing the solution to flow over the rim of the bath, after which it drains to a sink below. A computer VI panel enables the experimenter to select from up to 6 different taste stimuli or deionized H_2_O in the reservoir bottles and to set solution temperature (37°C) and flow rate (3 ml/sec). Solution temperature is monitored by a thermocouple located at the base of the bath.

### Procedure

Subjects who had not served previously in experiments in our laboratory participated in a training session in which they received instructions and practice in the use of the general version of the Labeled Magnitude Scale (gLMS) [18–20] to rate the intensity of taste sensations. Intensity ratings were made on a computer using custom data collection software.

#### Experiment 1

This experiment included 5 conditions to measure the effect of sucrose on the SWT: (1) Lac→H_2_O (lactisole followed by 5 water rinses); (2) Mix→H_2_O (lactisole + sucrose followed by 5 water rinses); (3) Suc→Lac→H_2_O (sucrose before lactisole followed by 4 water rinses); (4) Lac→Suc→H_2_O (lactisole before sucrose followed by 4 water rinses); and (5) Lac→H_2_O→Suc→H_2_O (lactisole and a water rinse before sucrose followed by 3 water rinses). Five pseudorandom orders were created so that each condition appeared first or last in one of the orders, and each subject was randomly assigned to an order. All 5 test conditions were presented in each of 2 sessions to obtain replicate data. Subjects remained blind throughout testing to the contents of the solutions and to the phenomenon of SWT. On the first trial of each condition subjects sipped the full sample into the mouth and swished it vigorously for 5 sec. Five seconds after expectorating the sample, and with no rinse between, the subjects sipped and vigorously swished the second sample for 10 sec. At the experimenter’s signal the subject expectorated the sample and immediately rated the peak intensity of sweetness, saltiness, sourness, and/or bitterness that was experienced while the sample was in the mouth. After 15 sec subjects were cued to sip the next sample, and the same procedure was repeated. After all 6 samples had been swished and rated, subjects rinsed repeatedly with dH_2_O during a 5-min break to ensure the SWT was no longer present when the next condition was tested.

#### Experiment 2

To test the generality of the effects of sucrose on the SWT, this experiment tested 5 additional sweeteners in 3 of the conditions of experiment 1: (1) Lac→H_2_O (lactisole followed by 5 water rinses); (2) Mix→H_2_O (lactisole + sweetener followed by 5 water rinses), and (3) Lac→Sweetener →H_2_O (lactisole before a sweetener followed by 4 water rinses). The timing was the same as experiment 1. All 3 conditions were presented in each of two testing sessions in which the participants received 2 or 3 of the 5 sweeteners in the first session and the remaining sweeteners in the second session. There were 5 condition and stimulus orders organized such that each sweetener (and lactisole) was presented first or last in one of the orders, and subjects were again randomly assigned to the orders. The procedures for sipping and swishing the samples was the same as experiment 1, as was the procedure for rating taste intensity.

After data collection was completed a control condition was added in which a new group of subjects rated the perceived intensity of sweetness for the same 5 sweeteners alone, without pre-exposure to lactisole or in mixture with lactisole (i.e., Sweetener→H_2_O rinses). These data were collected to determine whether residual sweetness is experienced during the 5 water rinses after initial exposure to the stimulus which might be consistent with differences in sweetener potency. Subjects were instructed to rate the maximum sweetness perceived during each exposure or water rinse, and were again assigned randomly to one of 10 stimulus orders.

#### Experiment 3

Exposure to stimuli in this experiment was limited to the tongue tip, and the stimuli were sampled in either flowing or still (non-flowing) solutions to compare the effects of passive vs active water rinsing on the SWT and on interactions between sweeteners and lactisole. The additional artificial sweetener neotame was tested together with sucrose to investigate further the apparent association between sweetener potency and suppression of the SWT; neotame has a potency that exceeds that of NHDC. The experiment had 7 conditions: (1) Baseline Sweetness, in which sweeteners were sampled in still solutions; (2) Sweetener + Lac, in which mixtures of lactisole and the sweeteners were sampled in still solutions; (3) Lac→Sweetener, in which lactisole was sampled before the sweeteners in still solutions; (4) Lac→H_2_O (still), in which lactisole was sampled in a still solution before dipping the tongue into still water; (5) Lac→H_2_O (*flowing*), in which lactisole was sampled in a still solution before dipping the tongue into *flowing* water; (6) Sweetener + Lac→H_2_O (*flowing*), in which mixtures of lactisole and individual sweeteners were sampled in still solutions before the tongue was dipped into *flowing* water; and (7) Lac→Sweeteners→H_2_O (*flowing*), in which the sweeteners were sampled after lactisole in still solutions before the tongue was dipped into a *flowing* rinse.

In all conditions the individual solutions and water rinses were sampled for 10 sec each. Subjects were instructed to extend the tongue between closed lips and to dip it into either weighing dishes containing the stimulus or a water rinse (still exposures) or into the tongue bath of the TFG (*flowing* exposures). At the end of the 10 sec interval the subject immediately lifted the tongue from the solution and either dipped it into the next solution or immediately began making intensity ratings of the sweetness, saltiness, sourness and bitterness experienced during tasting the last sample. Subjects made all intensity ratings with the tongue extended outside the mouth, and vigorously rinsed the whole-mouth with water between trials to washout residual stimulus. There were 6 pseudorandom orders of conditions to which subjects were randomly assigned, and all conditions were presented twice in a single test session to obtain replicate data.

#### Statistical analyses

Raw intensity ratings were converted to log_10_ values before analysis, consistent with the tendency for the ratings to be log-normally distributed on labeled magnitude scales [18, 19]. Repeated-measures ANOVAs (Statistica 13 Academic^™^, general linear model) were conducted first to identify main effects and interactions among factors, and specific contrasts of interest were further analyzed using the Tukey HSD test (p<0.05).

## Results

### Experiment 1

**Fig 1:**
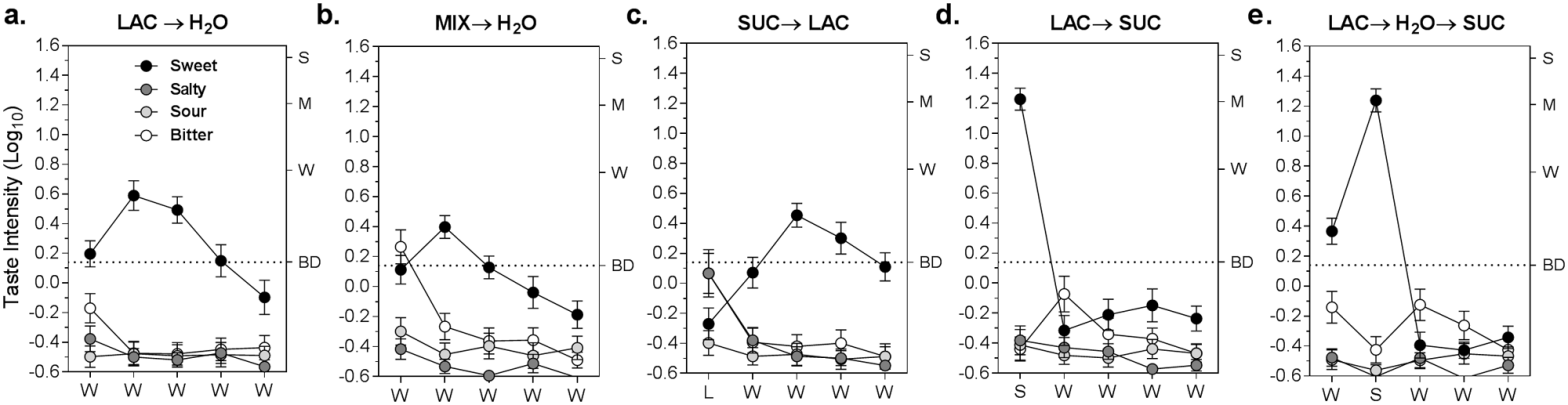
Effect of sucrose on the lactisole sweet water taste. The perceived intensity of sweetness (black circles), saltiness (dark gray circles), sourness (light gray circles), and bitterness (open circles) are shown for the 5 conditions of experiment 1: **(a)** LAC → H_2_O: lactisole followed by 5 water rinses; **(b)** MIX → H_2_O: lactisole + sucrose, followed by 5 water rinses; **(c)** SUC → LAC: sucrose before lactisole, followed by 4 water rinses; **(d)** LAC→ SUC: sucrose after lactisole, followed by 4 water rinses; and **(e)** LAC → H_2_O → SUC: sucrose after lactisole and 1 water rinse, followed by 3 water rinses. Letters on the x-axis of each graph represent water rinses (W), lactisole rinses (L), and sucrose rinses (S). Letters on the right y-axes represent semantic labels of sensation intensity on the gLMS: BD = barely detectable; W = weak; M = moderate, and S = strong. The dotted line at BD serves to highlight the near imperceptibility of most tastes other than sweetness, and the number of rinses over which the SWT was clearly perceived. Vertical bars indicate standard errors (SEs) of the log_10_ means.

Fig 1 shows the effect on the SWT of interactions between sucrose and lactisole at different time points. First, presenting lactisole before water (Fig 1a) resulted in an effectively pure SWT that increased in strength between the first and second rinses before gradually declining over the next 3 rinses. This time-course implied that lactisole continued to dissociate from the receptor throughout multiple water rinses.

When lactisole was mixed with sucrose (Fig 1b), the magnitude of the SWT was significantly reduced (main effect of Mix→H_2_O condition; F_(1,21)_ = 6.74, p<0.02), remaining above “barely detectable” for only the second water rinse. A significant condition x trial interaction (F_(8,84)_ = 2.73, p<0.05) reflected the greater difference between conditions over the intermediate rinses. The first water rinse following the mixture was also reported to have a barely detectable bitter taste that disappeared upon subsequent rinsing.

The SWT was also reduced in intensity when sucrose preceded lactisole (Fig 1c), though somewhat less than when sucrose was in mixture with lactisole. However, an analysis conducted on the equivalent 4 water rinses following lactisole for these 2 conditions (Mix→H_2_O vs. Suc→Lac→H_2_O) showed that the main effect of condition was just short of significance (F_(1,21)_ = 4.17, p = 0.054), and that there was no condition x trial interaction (F_(3,63)_ = 1.37, p = 0.26). As expected, sweetness was nil during exposure to lactisole, with only barely detectable bitterness and sourness reported, which were not perceived during the water rinses.

When sucrose followed lactisole (Fig 1d) subjects reported a “moderate” sweetness followed by complete suppression of the SWT (main effect of Lac→Suc→H_2_O condition, F_(1,21)_ = 64.0, p<0.00001). The SWT was also suppressed when sucrose was presented after the SWT had been established during the first water rinse (Fig 1e). It is noteworthy that the sweetness induced by sucrose was virtually identical in magnitude (1.24 vs. 1.25; Tukey HSD, p = 0.99) whether sucrose was tasted immediately after lactisole or after an intervening water rinse. The similar sweetness intensity in both conditions indicates that the sensitivity to sucrose recovered completely during both the first and second water rinses. This is a surprising result given the continuing growth of the SWT over the same 2 rinses (Fig 1a), which as was noted above implies that some amount of lactisole was still bound directly to or in close proximity to the receptors following the first rinse.

### Experiment 2

**Fig 2:**
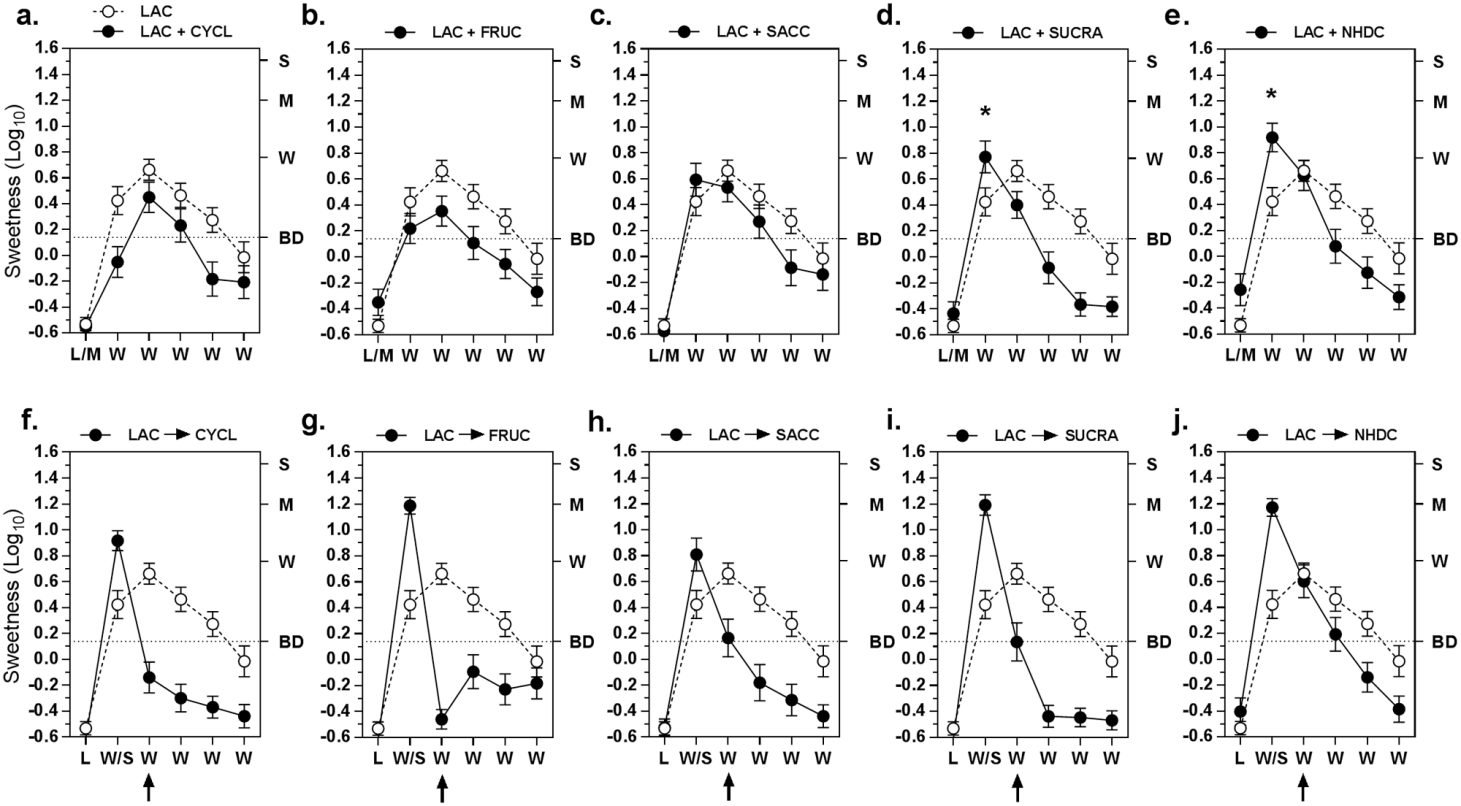
Effects of different sweeteners on the lactisole sweet water taste. Five different sweeteners (CYCL = cyclamate, FRUC = fructose, SACC = saccharin, SUCRA = sucralose, NHDC = neohesperidin dihydrochalcon) were presented in the 2 treatment conditions of experiment 2: in mixture with lactisole **(a-e)** or after lactisole **(f-j)**. The data for the lactisole SWT is shown in each graph (open circles) for comparison with the SWT produced in the treatment conditions (filled circles). In graphs **(a-e)** L/M on the x-axis indicates that in the treatment condition the mixture was presented on the first trial. Asterisks in graphs (d) and (e) indicate significantly higher SWT on the first water rinse after the mixture compared to the SWT after lactisole alone (Tukey HSD, p<0.05). In graphs **(f-j)** W/S indicates that in the treatment condition the sweetener was presented on the second trial. Thus in **(f-j)** the data on the second trial reflects the sweetness evoked by the sweetener, and the remaining 4 trials indicate the SWT. Arrows below the x-axis highlight the large differences in suppression of the SWT during the first water rinse after each sweetener compared to the second water rinse after lactisole alone. The horizontal dotted line in each graph indicates a “barely detectable” level of sweetness. Vertical bars are SEs of the log_10_ means.

An important question raised by the results of experiment 1 was whether the interactions between lactisole and sucrose reflected general properties of all agonists of the TAS1R2-TAS1R3 sweet taste receptor. Fig 2 displays the sweetness reported when 5 other sweeteners were presented in mixture with lactisole (Mix→H_2_O condition) prior to 5 water rinses (Fig 2a–2e), compared to when they were presented after lactisole (Lac→Sweetener→H_2_O condition) and prior to 4 water rinses (Fig 2f–2j). Shown for comparison is the SWT produced by lactisole prior to 5 water rinses (Lac→H_2_O condition; black circles), which replicated the intensity and time-course of the SWT found in experiment 1 (Fig 1a).

The data show that the sweetness of all 5 sweeteners was completely blocked when they were presented in mixture with lactisole. However, the strength and time-course of the subsequent SWT varied in accordance with the potency of the sweeteners. Similar to sucrose in experiment 1, cyclamate and fructose, the least potent of the artificial sweeteners, significantly suppressed the SWT throughout its time-course (Fig 2a and 2b). Separate ANOVAs comparing the Lac→H_2_O conditions to the respective Mix→H_2_O conditions confirmed there were main effects of condition for both sweeteners (cyclamate, F_(1,19)_ = 18.48, p<0.0005; fructose, F_(1,19)_ = 16.53, p<0.001), and an ANOVA that directly compared the SWT produced by the 2 mixtures found no main effect of stimulus and no stimulus x trial interaction. In contrast, mixing the higher-potency saccharin with lactisole (Fig 2c) did not reduce the sweetness of the first water rinse before attenuating sweetness on later rinses (condition x trial interaction; F_(4,76)_ = 5.72, p<0.0005), and the 2 highest potency sweeteners, sucralose and NHDC (Fig 2d and 2e), produced sweetness ratings during the first water rinse that were significantly higher than after lactisole alone (Tukey HSD, p<0.05) before sweetness declined over later trials (condition x trial interactions; sucralose, F_(4,76)_ = 14.40, p<0.00001; NHDC, F_(4,76)_ = 14.03, p<0.00001).

As was found in experiment 1 for sucrose, the sweetness of cyclamate and fructose was not blocked when they followed a lactisole rinse (Lac→Sweetener→H_2_O conditions; Fig 2f–2j), and both abolished the SWT. However, sweetness remained barely perceptible on the first water rinse following either saccharin or sucralose (Fig 2h and 2i) and was not inhibited after NHDC (Fig 2j). These differences mirrored those found for lactisole-sweetener mixtures and also appear to be correlated with agonist potency. An overall ANOVA showed that when collapsed across sweeteners the SWT was significantly suppressed (main effect of sweetener; F_(4,76)_ = 1.90, p = 0.12), and that there was a significant sweetener x time interaction (F_(20,380)_ = 5.50, p<0.00001) due to the large differences in the suppression of the SWT over time.

**Fig 3:**
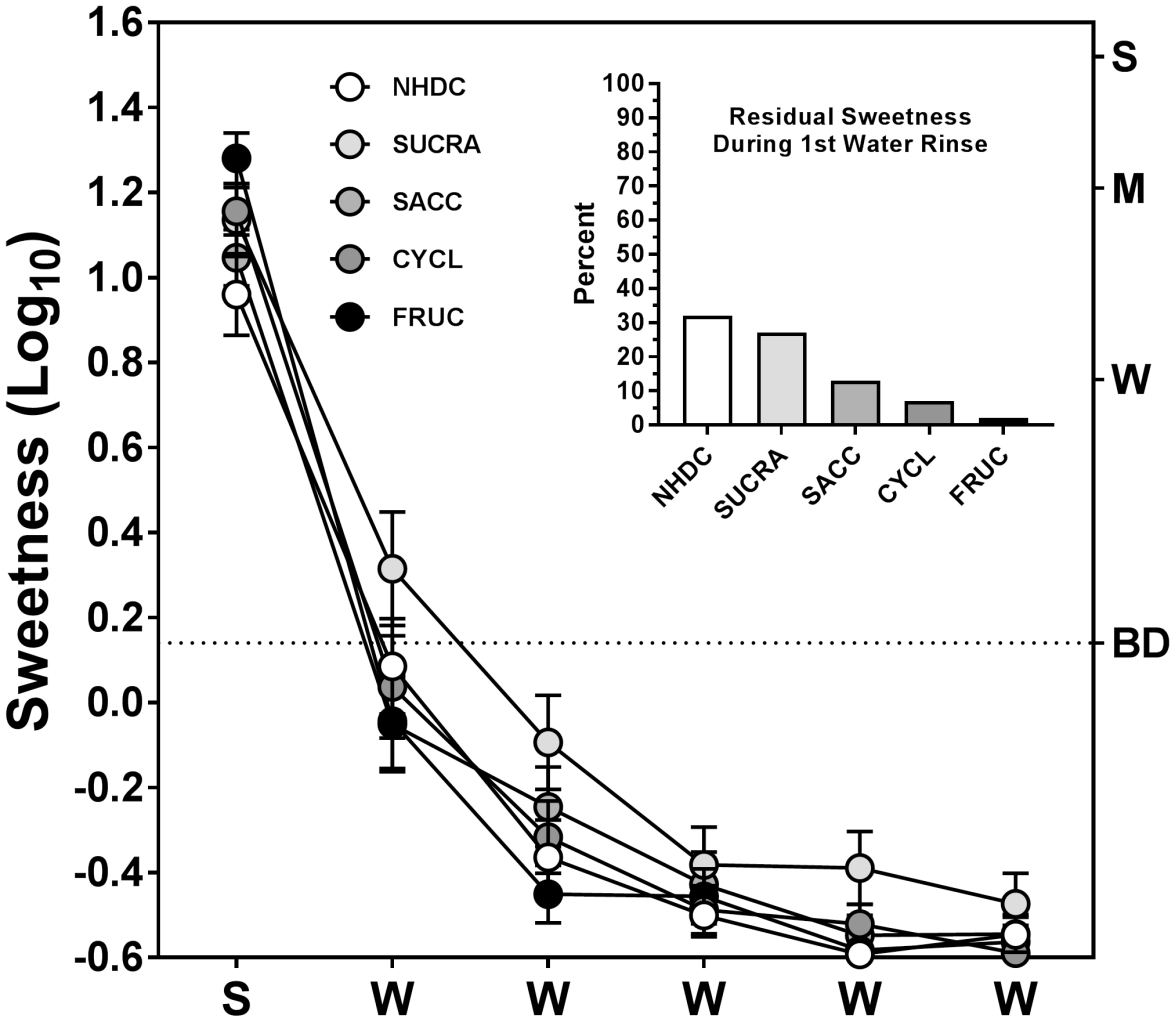
Decay in sweetness produced by sweeteners alone across water rinses. Main graph: Peak sweetness ratings for the 5 sweeteners of experiment 2 when presented alone and during 5 water rinses. S and W’s on the x-axis indicate exposures to the sweeteners and water rinses, respectively. The horizontal dotted line indicates a “barely detectable” level of sweetness; vertical bars are SEs of the log_10_ means. Inset: residual sweetness during the first water rinse expressed as a percentage of the initial sweetness reported for each of the sweeteners.

The data of Fig 3 show the initial intensities of sweetness produced by the 5 sweeteners alone and the decline in rated sweetness over 5 water rinses. The data were collected from subjects who did not participate in the main experiment. A repeated-measures ANOVA limited to the initial sweetness ratings found that sweetness intensity varied significantly across stimuli (main effect of stimulus; F_(4,112)_ = 4.87, p<0.005), with the effect driven by a significant difference between the sweetnesses of fructose and NHDC (Tukey HSD, p<0.05). A between-subjects ANOVA that compared the present data to data ratings of initial stimulus sweetness obtained from the subjects who participated in the main experiment found no significant difference (Stimulus x Group interaction; F_(4,188)_ = 0.46, p = 0.77), with both groups reporting fructose and NHDC as having the highest and lowest sweetness, respectively. A repeated-measures ANOVA on the time-course of sweetness data in Fig 3 revealed a significant stimulus x water rinse interaction (F_(20,560)_ = 2.58, p<0.0005), indicating that sweetness declined at different rates across stimuli. This effect is seen more clearly in the inset in Fig 3 which shows the sweetness perceived during the first water rinse as a percentage of the initial sweetness rating given to each stimulus. This residual sweetness was highest for the 2 highest-potency sweeteners, NHDC and sucralose, and lesser amounts of sweetness were reported for the other 3 sweeteners that were also consistent with their relative potencies.

### Experiment 3

**Fig 4:**
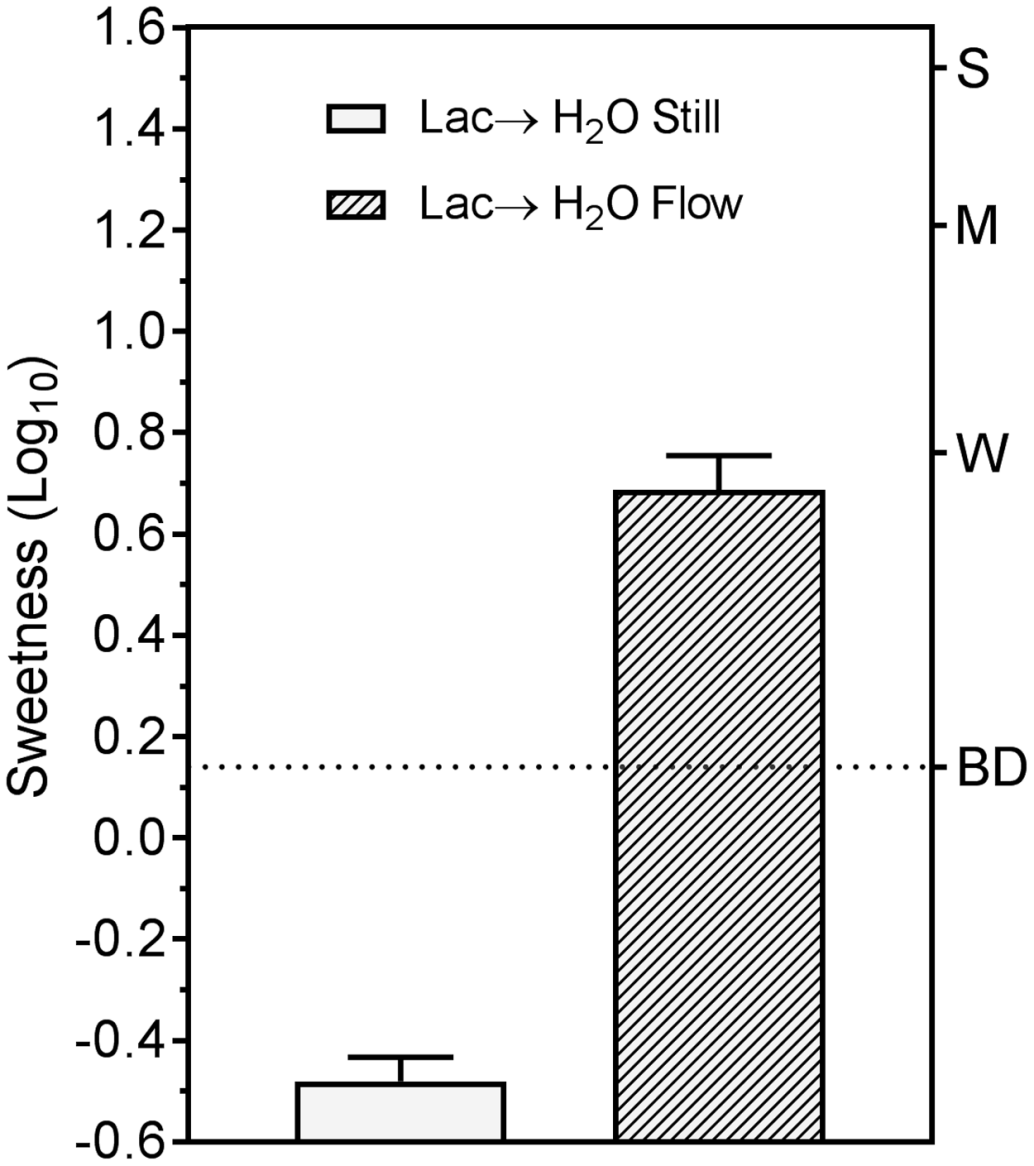
Lactisole sweet water taste requires flowing water rinses. Shown are log_10_ ratings of the lactisole SWT experienced on the tongue tip in experiment 3 under conditions of *Still* vs. *Flowing* water rinses. The dotted line indicates the level of “barely detectable” sweetness. Vertical bars are the SEs of the log_10_ means.

**Fig 5:**
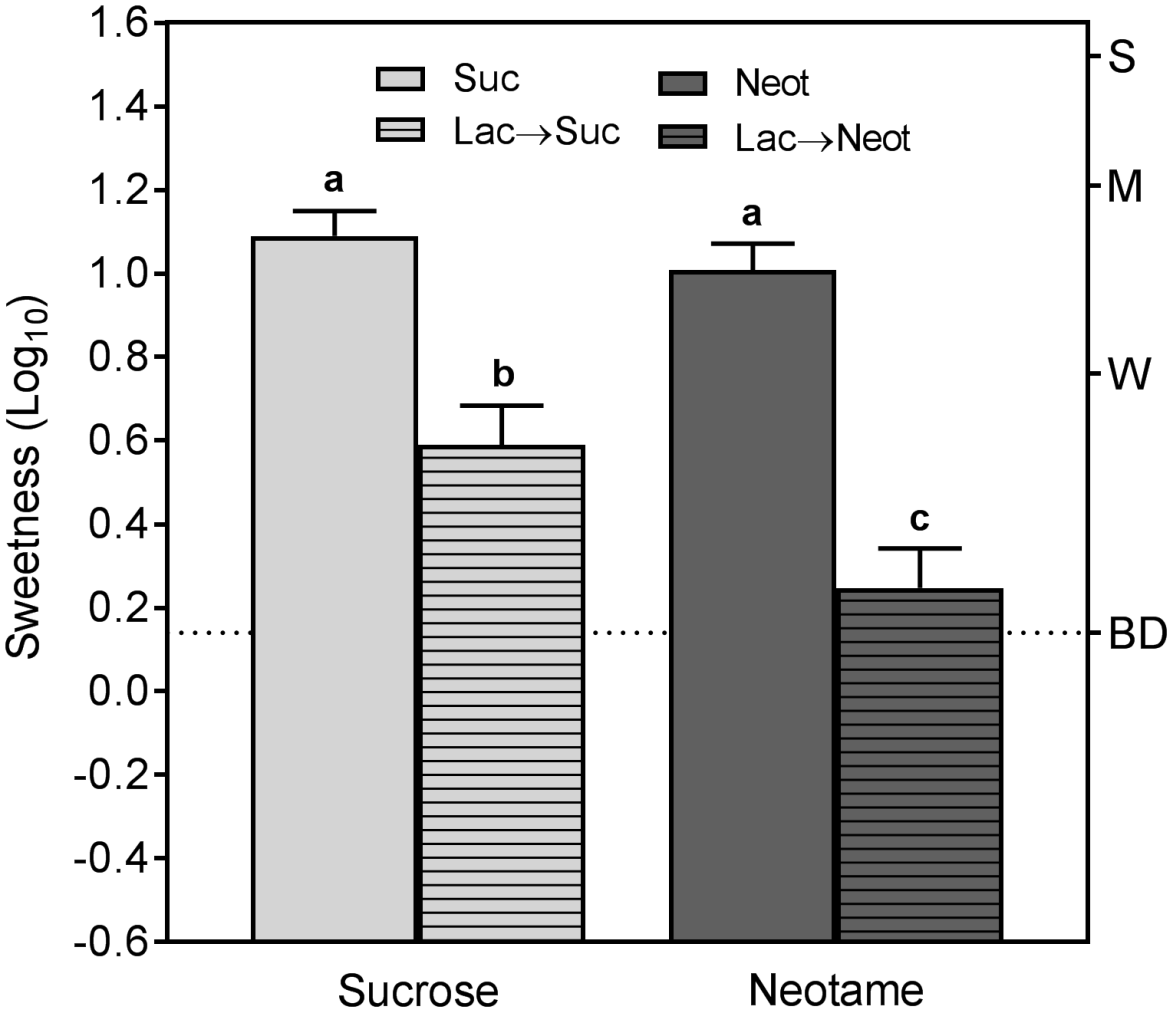
Pre-exposure to lactisole suppresses the sweetness of sucrose and neotame in ‘still’ solutions. Shown are log_10_ ratings of the sweetness of *still* solutions of sucrose or neotame perceived on the tongue tip when the solutions were sampled alone or following exposure to a *still* solution of lactisole, in which rinsing of lactisole is assumed to be incomplete and therefor results in partial suppression of sweetness. Different letters over the bars indicate significant differences in sweetness. The dotted line indicates the level of “barely detectable” sweetness. Vertical bars are the SEs of the log_10_ means.

As expected based on our preliminary observations, lactisole produced a perceptible SWT on the tongue tip only when the tongue was dipped into flowing water (Fig 4). Consistent with this result, Fig 5 shows that when still solutions of sucrose or the high-potency sweetener neotame were sampled after lactisole (Lac→Sweetener conditions), sweetness was suppressed compared to when the sweeteners were sampled alone. These results imply that mere exposure to still water for 10 sec does not lead to rapid dissociation of lactisole from sweet taste receptors. However, in this condition the suppression of sweetness by lactisole was greater for neotame than it was for sucrose. A repeated measures ANOVA on the data in Fig 5 indicated there was a significant stimulus x condition interaction (F_(3,33)_ = 46.35, p<0.00001), and Tukey HSD tests confirmed that the sweetness evoked by sucrose and neotame before lactisole did not differ significantly (p = 0.74), whereas after lactisole the sweetness of neotame was reduced more than the sweetness of sucrose (p<0.0005).

**Fig 6:**
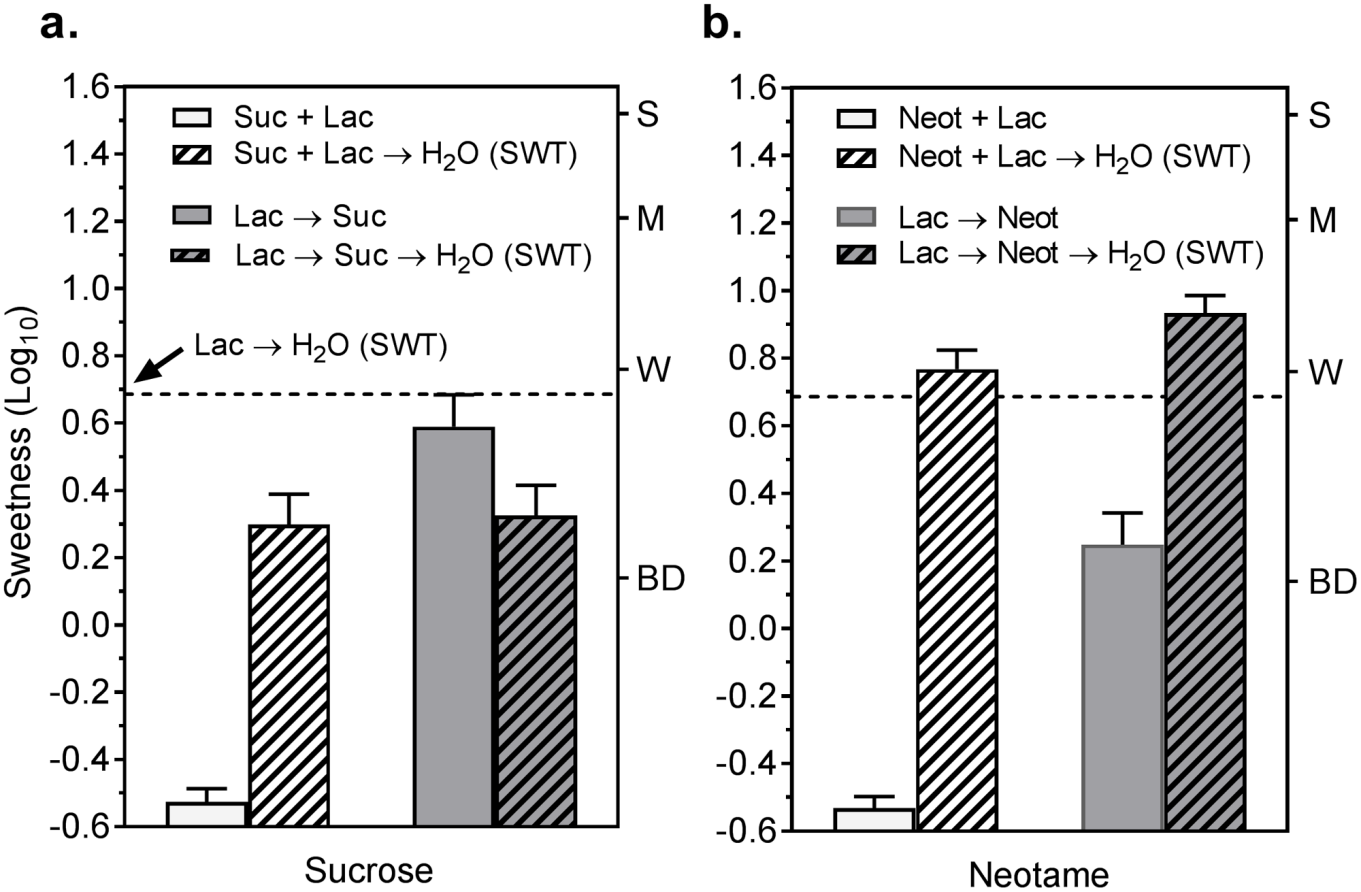
Lactisole sweet water taste is unaffected by prior sweet receptor activation. Log_10_ sweetness ratings are shown for solutions of **(a)** sucrose and **(b)** neotame sampled with the tongue tip in 4 conditions: Suc or Neot + Lac: a *still* lactisole + sweetener mixture (white bars); Lac→Suc or Neot: a *still* solution of lactisole before a *still* solution of the sweetener (gray bars); Suc or Neot + Lac → H_2_O: a *still* sweetener + lactisole mixture followed by a *flowing* water rinse (hatched white bars); and Lac→Suc or Neot→H_2_O: a *still* solution of lactisole before a *still* solution of the sweetener followed by a *flowing* water rinse (hatched gray bars). Shown for comparison is the SWT produced by a *flowing* water rinse after lactisole alone (dashed line). Vertical bars are the standard errors of the log_10_ means (SEMs).

Fig 6 shows the intensity of the SWT perceived when water was presented following mixtures of lactisole and either of the 2 sweeteners (Sweetener+Lac→H_2_O), or following sequential exposure to lactisole and the sweeteners (Lac→Sweetener→H_2_O). The SWT after lactisole alone (dashed line) is shown for comparison. In both conditions the SWT was suppressed by sucrose (Fig 6a) but not by neotame (Fig 6b). These results on the tongue tip are consistent with those found for the first water rinse in the whole-mouth conditions of experiment 2, including the inverse association between suppression and potency. Specifically, like fructose and cyclamate, sucrose suppressed the SWT, and like sucralose and NHDC, neotame initially enhanced it. The data also show that for both sucrose and neotame, the magnitude of the SWT (hatched bars) was approximately the same whether sweetness was perceived (Lac→Suc and Lac→Neot) or not perceived (Suc+Lac and Neot+Lac) prior to exposure to flowing water

## Discussion

The most notable finding of this study is that agonists of the sweet taste receptor can modulate the lactisole SWT when presented before, during, or after lactisole. The first question to consider is whether the existing model of the mechanism of the SWT can explain these effects. Galindo-Cuspinera et al. [8] have proposed that the SWT occurs when “…removing lactisole induces a coordinated shift of the receptors from the high-energy, inhibited state to the constitutively active state” (p. 356). However, the differential effects of sweeteners on the SWT in mixture as well as before and after lactisole suggest the possibility that additional factors may be involved in the development and modulation of the SWT.

First, it is not clear that when lactisole dissociates from TAS1R2-TAS1R3 that the receptor only returns to its constitutively active state. *In vitro* Ca^++^ imaging data from the Galindo-Cuspinera et al. [8] study shows that expression of the receptor in HEK293 cells led to a higher basal intracellular Ca^++^ level, which was then reduced below baseline levels by 3-mM lactisole (p. 356, Fig 4g). However, other data from the same study (Fig 4d–4f) show that following either lactisole or high (blocking) concentrations of saccharin or acesulfame-K, water rinses caused sharp phasic increases in Ca^++^ indistinguishable from excitatory increases produced by 3-mM acesulfame-K or saccharin, which in humans evokes weak-to-moderate sweetness (see p. 354, Fig 1c); also [21]. These data suggest that either (1) returning intracellular Ca^++^ to baseline levels is sufficient to activate receptor cells to a degree equivalent to that produced when an agonist binds to the receptor, or (2) that dissociating lactisole activates the cells directly. We can find no other published evidence that dissociation of inhibitory ligands can activate GPCRs. However, as ligands dissociate, receptor conformation must revert to its prior state(s). However, it is notable that for other family C GPCRs, compounds with binding sites residing in the TMD can act as both as positive allosteric modulators (PAMs), or even agonists [22]. What may be unique to lactisole and other sweet taste inhibitors which bind in the TMD of hT1R3 [11] is that this conformational reversion following their removal is sufficient to activate the receptor. Jiang et al. [11] proposed that lactisole blocks the TAS1R2-TAS1R3 receptor by “…binding to TM helixes 3, 5, and 6 of hT1R3 so as to restrict movement required for conversion to the active state” (p. 15246). We speculate that the mechanical unblocking of the TM helixes may cause sufficient movement of the helixes [23] to activate the G-protein and initiate transduction [24, 25]. This process could be similar to the mechanism of rhodopsin activation where light-dependent conversion of 11-cis-retinal (a covalent inverse agonist) to all-trans-retinal removes the inhibitory effects of 11-cis-retinal and enables the receptor to initiate G-protein-dependent signal transduction [26]. Whichever of these mechanisms underlies the SWT, it was surprising to find that sweetness continued to increase during the second water rinse before slowly declining over 5 rinses. If it is assumed the second rinse unblocks additional receptors which add to the total afferent outflow, either via a return to a constitutively active state or direct activation of the receptor transduction cascade. However, this interpretation requires that lactisole remains bound to TAS1R2-TAS1R3 through multiple water rinses. Experiments on the binding potential of lactisole would help to answer this question along with a psychophysical experiment that tested a wide range of lactisole concentrations.

The present results also show that sweeteners can modulate the SWT when co-administered with lactisole. The possibility that these effects result from direct interactions between proximal binding sites of lactisole and agonist is not supported by the data: cyclamate, NHDC, and lactisole all bind in the TMD of TAS1R3 [11, 27–29], but cyclamate suppressed sweetness across all water rinses (Fig 2a) whereas NHDC enhanced the SWT before suppressing it (Fig 2e). Sucralose, which binds in the Venus Fly Trap domain (VFD) of TAS1R2 [30], produced a pattern of SWT enhancement and suppression similar in magnitude and timing to that produced by NHDC. In addition, in experiment 3, neotame, which also binds in the VFD of TAS1R2 [31], also tended to enhance sweetness during the water rinse (Fig 6b). These results might be explained by allosteric modulation. Ligands of GPCRs have the potential to inhibit (negative allosteric modulators; NAMs) or enhance (positive allosteric modulators, PAMs) [32, 33] the excitatory effects of agonists by producing conformational changes across receptor domains and between monomers [34]. Allosteric modulation has been studied most extensively in non-gustatory GPCRs (e.g. [35, 36, 37]), but PAMs have been proposed to explain how ligands that do not taste sweet can enhance sensitivity to receptor agonists that do taste sweet [15–17]. Because lactisole acts as an inverse agonist [8, 9], sweeteners can bind to and produce conformational changes within the receptor even as their ability to excite it is blocked. Whether an agonist can suppress or enhance the lactisole SWT may depend on how the conformational change it produces interacts with the conformational change caused by lactisole’s dissociation from the TMD. The fact that in most C GPCRs PAMs bind to the TMD [38] demonstrates the potential for conformational interactions of this kind to take place. The abrupt and complete block of the SWT by sweeteners sampled after exposure to lactisole was also agonist-dependent and might also be explained by allosteric interactions, as conformational changes produced by agonists may interact with conformational changes produced by dissociating lactisole. Although lactisole can suppress the excitatory effects of agonists both in mixture (Fig 6) and when it appears to remain partially bound to the receptor in still, aqueous solutions (Fig 5), the results also show that during a kinetic water rinse, agonists can evoke sweetness as soon as lactisole begins to dissociate. Here too, lesser suppression of the SWT on the first water rinse by high potency agonists might be due to a fraction of the sweetener remaining bound to the receptor as rinsing continues. Conversely, it is possible the higher potency sweeteners have a higher affinity for the receptor which stabilizes different tertiary receptor conformations and that these conformational differences alter the manner in which the allosteric modulator (lactisole) interacts with the receptor complex, which has been observed for other GPCR systems [39].

The findings that mixing sucralose or NHDC with lactisole initially enhanced the SWT, and that the SWT was suppressed least during the first water rinse following NHDC, further suggest that the relative potency of the sweeteners, and thus possibly their affinity for the TAS1R2-TAS1R3 receptor, might be a factor. Based on concentrations that produce approximately equivalent sweetness, the potencies of the 5 stimuli of experiment 2 are ordered as follows: NHDC > sucralose >> saccharin > cyclamate > fructose. This order correlates with the range of suppression and enhancement of sweetness during the first water rinse (Fig 2a–2e), especially with respect to NHDC and sucralose in experiment 2 and neotame in experiment 3. Those sweeteners have potencies that are approximately an order of magnitude greater than the other agonists and are the only ones that significantly enhanced sweetness during the first water rinse. Higher potency sweeteners are more likely to remain bound during the first water rinse which could augment activation of receptors as they continue to be unblocked by lactisole. Higher potency sweeteners might also remain in the lipid bilayer of the oral epithelium, making them available to bind to unblocked receptors during the water rinses [40], The data in Fig 3 supports both of these possibilities for the stimuli of experiment 2 by showing that during the first water rinse, residual sweetness was perceived more strongly for NHDC and sucralose than for the other 3 sweeteners, and that for all 5 stimuli residual sweetness was inversely related to potency. This ordering of stimuli also agrees well with the psychophysical results for suppression and enhancement of sweetness following mixtures with lactisole (Fig 2a–2e), but somewhat less so for the suppression of the SWT after lactisole (Fig 2f–2j). Other receptor mechanisms in addition to allostery and sweetener potency must also be considered as possible factors in suppression of the SWT by sweeteners. For example, stimulation of TAS1R2-TAS1R3 by a sweetener might reduce the sensitivity of the receptor via adaptation or desensitization. However, a recent study from this laboratory [21] found that pre-exposure to a sweetener in 37°C solutions does not extinguish the sweetness of a second exposure to the same stimulus (self-adaptation), and there is no evidence from the in vitro data of Galindo-Cuspinera et al. [8] (see their Fig 4d–4f) that heterologously expressed TAS1R2-TAS1R3 became less sensitive after activation by saccharin or acesulfame-K. Results from experiment 3 of the present study (Fig 6) also show that the SWT was not reduced when sweetness was perceived (and thus the receptors activated) prior to a water rinse (i.e., Lac→Suc→ H_2_O; Lac→Neot→H_2_O). Although the sweetness evoked following lactisole was not as strong as when the sweeteners were presented alone, if shifting receptors to a less sensitive state following stimulation plays a key role in suppressing the SWT, some reduction in the SWT would be expected.

Desensitization of TAS1R2-TAS1R3 must also be considered, since hyperosmotic concentrations of sucrose (≥32 mM) can block endocytosis in GPCRs, thereby preventing phosphorylation, recycling, and resensitization [41]. Because the concentrations of sucrose, fructose, and cyclamate were all hyperosmotic, suppression of the SWT by those 3 stimuli could in principle result from this mechanism. However, the rate of endocytosis in GPCRs is measured on a time scale of minutes rather than seconds [42, 43], and pharmacological studies using hypertonic sucrose to block endocytosis typically employ incubation periods of several minutes [44, 45]. Hypertonicity and endocytosis therefore seem unlikely to have played a significant role in the rapid suppression of SWT that we observed.

In experiment 3, perception of the SWT when the tongue tip was dipped into flowing but not still water demonstrates that mere exposure to water fails to liberate lactisole from taste receptors at a sufficient rate to evoke sweetness. It is likely that during the application of still water, any unbound lactisole can simply re-associate with the receptor complex and thus prevent SWT from occurring. The application of kinetic rinsing creates a scenario of disequilibrium between bound and free lactisole, allowing unbound lactisole to be removed and ultimately leading to the perception of SWT. This finding may also explain an anecdotal but consistent observation that was made during preliminary testing for this study: as saliva began returning to the mouth between water rinses, a sweet “aftertaste” developed that was localized primarily in the back of the mouth. Because salivary ducts of the parotid gland are located high on the buccal walls near the back of the mouth [46], saliva from those ducts tends to flow down the buccal wall and over the foliate taste papillae that are located along the posterior edges of the tongue. This kinetic rinsing action is less likely to occur in the front of the mouth, where saliva entering the mouth via the sublingual and submandibular ducts [47] bathes fungiform taste papillae in a pool of saliva.

The additional finding that the sweetness of both sucrose and neotame was partially suppressed following pre-exposure to lactisole in experiment 3 (Fig 5) provides further evidence that compared to whole mouth rinsing, dipping the tongue into a still aqueous solution leaves significantly more bound lactisole. The stronger suppression of the sweetness of neotame also indicates that lactisole’s blocking effect differs across receptor agonists, a finding first reported by Schiffman et al. [48]. Using much lower concentrations of lactisole than in the present study (250 and 500 ppm vs 8mM, or 1,745 ppm), those researchers found that when presented in mixture, lactisole did not block the sweetness of all sweeteners, including NHDC or the protein sweetener thaumatin. At the time of their study lactisole was believed to act as a competitive antagonist [49], which led Schiffman et al. to interpret their results as evidence of multiple sweet taste receptors. Given that lactisole acts as an inverse agonist [6–9, 27], the results of Schiffman et al., and those of experiment 3 in which lactisole was presented before sucrose and NHDC, could be explained if lactisole’s ability to block receptor activation varies depending on the conformational changes produced by agonists. This interpretation is consistent with the idea that lactisole mechanically locks the receptor in an inactive state [11], an action that may not be equally effective against conformational changes produced by different agonists. We speculate that dipping the tongue tip into still, aqueous solutions after lactisole reduced the amount of bound lactisole to a level that exposed differential blocking of the excitatory effects of sucrose and neotame.
